# Research on Denoising Methods for Magnetocardiography Signals in a Non-Magnetic Shielding Environment

**DOI:** 10.3390/s25196096

**Published:** 2025-10-03

**Authors:** Biao Xing, Xie Feng, Binzhen Zhang

**Affiliations:** 1 Key Laboratory of Instrumentation Science & Dynamic Measurement, Ministry of Education, North University of China, Taiyuan 030051, China; s202106104@st.nuc.edu.cn; 2 Suzhou Cardiomox Co., Ltd., Suzhou 215000, China; xie.feng@cardiomox.cn

**Keywords:** magnetocardiography (MCG), variational mode decomposition (VMD), un-magnetically shielded, arithmetic optimization, wavelet threshold, signal denoising

## Abstract

Magnetocardiography (MCG) offers a noninvasive method for early screening and precise localization of cardiovascular diseases by measuring picotesla-level weak magnetic fields induced by cardiac electrical activity. However, in unshielded magnetic environments, geomagnetic disturbances, power-frequency electromagnetic interference, and physiological/motion artifacts can significantly overwhelm effective magnetocardiographic components. To address this challenge, this paper systematically constructs an integrated denoising framework, termed “AOA-VMD-WT”. In this approach, the Arithmetic Optimization Algorithm (AOA) adaptively optimizes the key parameters (decomposition level K and penalty factor α) of Variational Mode Decomposition (VMD). The decomposed components are then regularized based on their modal center frequencies: components with frequencies ≥50 Hz are directly suppressed; those with frequencies <50 Hz undergo wavelet threshold (WT) denoising; and those with frequencies <0.5 Hz undergo baseline correction. The purified signal is subsequently reconstructed. For quantitative evaluation, we designed performance indicators including QRS amplitude retention rate, high/low frequency suppression amount, and spectral entropy. Further comparisons are made with baseline methods such as FIR and wavelet soft/hard thresholds. Experimental results on multiple sets of measured MCG data demonstrate that the proposed method achieves an average improvement of approximately 8–15 dB in high-frequency suppression, 2–8 dB in low-frequency suppression, and a decrease in spectral entropy ranging from 0.1 to 0.6 without compromising QRS amplitude. Additionally, the parameter optimization exhibits high stability. These findings suggest that the proposed framework provides engineerable algorithmic support for stable MCG measurement in ordinary clinic scenarios.

## 1. Introduction

Cardiovascular diseases have long been one of the major causes of death globally and continue to exhibit a high incidence and substantial healthcare burden [1]. Existing clinical diagnostic approaches primarily rely on electrophysiological testing (ECG) and imaging modalities (Ultrasound/CT/MRI). However, these methods are constrained by limitations including variability in electrode contact quality, differences in tissue conductivity, exposure to radiation, contraindications for magnetic resonance imaging, and reduced sensitivity for detecting early occult lesions.

Electrocardiogram (ECG) is the fundamental diagnostic tool for cardiovascular diseases. It is used for cardiac function assessment and preliminary screening by recording myocardial electrical activity [2]. However, the contact-based acquisition of its body surface electrodes is susceptible to anatomical differences, electrode–skin impedance, and environmental noise, which can lead to waveform distortion and reduced accuracy. Moreover, the sensitivity of ECG in detecting early myocardial ischemia is limited, with approximately 40% of initial cases reported as missed diagnoses [3].

Coronary angiography (CAG) is considered as the clinical diagnostic standard due to its excellent vascular imaging [4]. It obtains three-dimensional information of coronary arteries by injecting a contrast agent through a catheter under X-ray fluoroscopy, providing accurate anatomical basis for treatment decisions. Nevertheless, it is an invasive procedure associated with risks of allergic reactions to iodine-based contrast agents [5] and puncture-related complications. CAG’s utilization is further limited for patients with renal insufficiency, thus promoting the exploration of non-invasive alternative diagnostic approaches.

Magnetic resonance imaging (MRI) and Computed Tomography (CT) each offer distinct advantages in the assessment of cardiac structure and function: MRI has high soft tissue resolution and can display myocardial morphology, contraction, and hemodynamic characteristics [6]; CT coronary angiography has high spatial resolution, which is beneficial for the assessment of stenosis, calcification, and anatomical variations [7]. However, MRI is contraindicated for patients with metal implants [8], and the ionizing radiation of CT brings cumulative risks to those who need repeated examinations [9]. Therefore, the development of new detection technologies that are safer, more convenient, and cost-effective is of great significance for improving the early detection rate and diagnostic accuracy of cardiovascular diseases.

Myocardial cells form macroscopic current dipoles during depolarization and repolarization. According to the Biot-Savart Law, these currents generate an extremely weak magnetic field around the thoracic cavity. As a non-invasive diagnostic technology based on the bio-magnetoelectric effect, magnetocardiography (MCG) obtains information on cardiac function and structure by detecting such weak magnetic fields produced by cardiac electrical activity. This magnetic field can penetrate tissues and bones and can be measured without skin contact, which is different from the way ECG measures potential differences. Due to the extremely low magnetic permeability of the human body, magnetocardiographic signals can be transmitted to the outside with low loss, resulting in richer information and more accurate localization of electrical activity [10,11].

**Figure 1:**
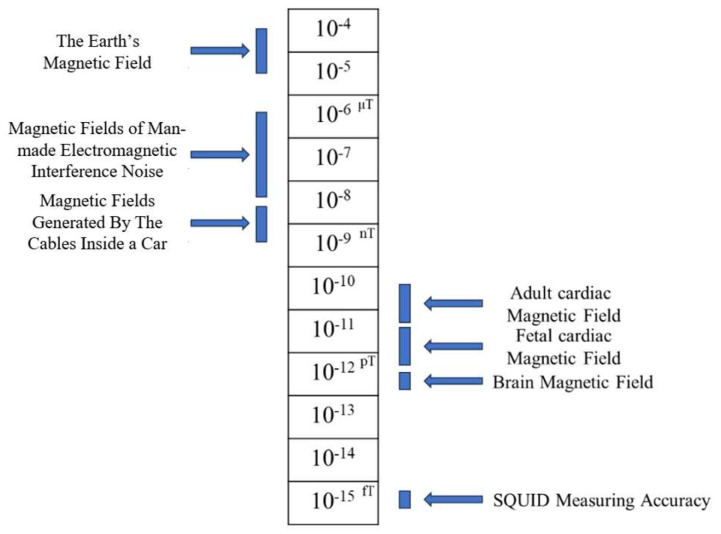
Comparison chart of common magnetic fields.

However, as shown in Figure 1, the intrinsic signal intensity of magnetocardiography is extremely low (about 20–100 pT) [12,13], necessitating the use of highly sensitive and precise sensors for reliable detection and collection. Currently, the Superconducting Quantum Interference Device (SQUID) has become the preferred equipment due to its magnetic field resolution at the femtoTesla level [14].

**Figure 2:**
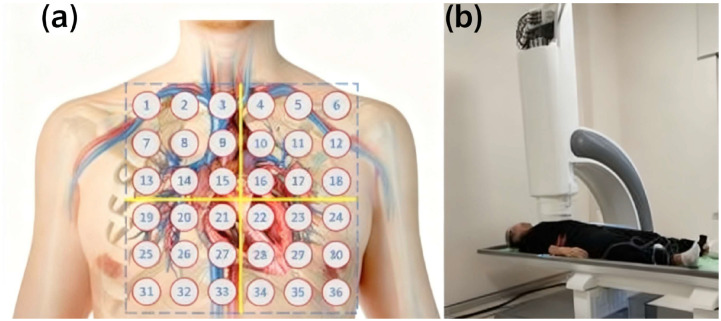
(**a**) Measurement points of magnetocardiography; (**b**) Measurement process of 9-channel magnetocardiography.

Nevertheless, there is a serious risk that the detection of the extremely weak MCG signals faces interference from environmental magnetic fields. Conventional MCG detection requires specialized magnetically shielded rooms [15], which results in high equipment costs and poor portability, thereby restricting its clinical application. To overcome these limitations, new MCG systems that operate without a magnetically shielded room have been developed [16,17]. By improving signal processing algorithms, these systems enable reliable measurements in standard clinical environments while reducing equipment costs, thus creating more opportunities for the popularization and application of MCG technology (Figure 2). Accordingly, achieving reliable magnetocardiographic detection and robust denoising in a non-magnetically shielded environment presents a critical technical proposition to promote the popularization of MCG. This paper addresses this challenge through conducting methodological investigations into signal acquisition, denoising, and reconstruction.

Since Laennec’s invention of the stethoscope, clinical cardiac diagnostics have evolved from physical examination to electrophysiology, and more recently, to image fusion [18,19]. ECG is well established for detecting rhythm abnormalities but remains limited in three-dimensional origin localization and characterization of epicardial repolarization abnormalities. Ultrasound presents advantages of real-time imaging and cost-effectiveness, but it is highly dependent on acoustic windows and operator expertise. CT and MRI provide high-resolution structural imaging capabilities, yet their clinical use is restricted by radiation exposures or contraindications.

The analysis of MCG signals provides complementary information on both the electrical and magnetic activities of the heart. By constructing magnetic field distribution maps above the cardiac region and further analyzing their characteristics, the underlying current vector dynamics can be inferred. Owing to the stronger penetrability of magnetic fields and their reduced sensitivity to tissue conductivity inhomogeneity, MCG is theoretically more suitable for the localization and spatial reconstruction of repolarization abnormalities, thereby offering additional value for comprehensive cardiac assessment [20,21,22,23].

Achieving high-precision capture and feature inversion of magnetocardiographic signals is essential in improving the reliability of heart disease diagnosis, with the critical challenge being the effective suppression of background noise interference on weak bio-magnetic signals. Since the intensity of magnetocardiographic signals is only about one-thousandth of that of environmental magnetic noise, interferences such as geomagnetic field fluctuations and electromagnetic radiation from equipment can easily mask effective physiological information, leading to signal distortion and even potential misjudgments.

Early studies primality employed traditional digital filtering techniques for noise reduction. Although such methods can partially eliminate high-frequency noise, they present significant limitations in practical applications. When the noise frequency band (such as 50 Hz or 60 Hz power frequency interference) overlaps with the characteristic frequency band of magnetocardiographic signals, conventional frequency-domain filtering struggles to achieve effective separation. This not only leaves substantial residual noise but may also compromise the signal waveform due to the steep cutoff characteristics of the filter, resulting in artifacts like distortion of the QRS complex morphology, which ultimately affects the accuracy of pathological feature extraction [24]. To solve issuers of power frequency interference and baseline drift in magnetocardiographic signals, researchers have successively explored various signal processing methods. In the early stage, high-order Infinite Impulse Response (IIR) filters were applied for noise suppression [25]. While they offer high computational efficiency and low resource occupation, their nonlinear phase response will distort the time-domain characteristics of the signal, impairing the analysis of pathological waveforms. To improve the noise reduction accuracy, the wavelet threshold (WT) decomposition methods were introduced to realize multi-scale signal analysis [26]. However, their effectiveness is highly dependent on the selection of wavelet basis functions, and different basis functions have significant differences in the ability to retain characteristic components such as the QRS complex. The subsequently developed Empirical Mode Decomposition (EMD) technology directly extracts the intrinsic mode of the signal through an adaptive decomposition mechanism [27], avoiding the limitations of preset basis functions, but it is prone to mode mixing during the decomposition process, resulting in ambiguous physical meaning of signal components. To improve decomposition stability, the Ensemble Empirical Mode Decomposition (EEMD) method enhances anti-mixing ability by adding white noise sequences [28], but the problem of noise residue still causes the loss of signal-to-noise ratio of the reconstructed signal, restricting the improvement of diagnostic accuracy.

In recent years, Variational Mode Decomposition (VMD), a new signal processing method based on variational optimization theory, has been proposed and has shown superior performance in multiple biological signal denoising tasks [29]. Studies have applied VMD to the denoising of ECG signals [30,31], demonstrating its good adaptive decomposition ability and robustness. However, as magnetocardiography (MCG) is an emerging technology, the application of VMD in MCG signal denoising is still in the exploratory stage, lacking systematic research and engineering verification. Independent Component Analysis (ICA) is a common method that decomposes multivariate signals into statistically independent sub-signals. However, determining which of the separated independent components belongs to real MCG signals and which are artifacts or noise usually relies on manual experience for judgment and screening. Such human intervention not only increases the subjectivity and uncertainty of signal processing but also significantly prolongs the data processing time, thereby limiting the promotion and practicality of the ICA method in clinical applications of MCG [32,33].

Based on the above situation, this work proposes an AOA-VMD-WT denoising framework for unshielded environments:

1. Using AOA to adaptively optimize VMD parameters (K, α), and then implement modal frequency perception rules and wavelet threshold/baseline correction;

2. To prevent the optimization algorithm from falling into a local optimum, the Arithmetic Optimization Algorithm (AOA) has been optimized and improved.

## 2. Materials and Methods

### 2.1. Data Sources

The MCG dataset used in this study was collected at Suzhou Dushu Lake Hospital, sampled at 1000 Hz. It consists of 9-channel MCG signals recordings from 4 regions (36 sets of MCG signal data in total) along with synchronized acquisition of ECG signals (4 sets of ECG signal data in total). The data were collected using a 9-channel Cardiomox MCG device manufactured by Suzhou Cardiomox Co., Ltd. (Suzhou, China). Data were manually classified as healthy or pathological based on coronary angiography. The data was run in the MATLAB 2021b software. The hardware configuration of the equipment used is as follows: the Central Processing Unit (CPU) model is Intel Core i5-12600KF, the Graphics Processing Unit (GPU) model is NVIDIA GeForce RTX 5060 Ti, and the Operating System (OS) is the 64-bit Windows 11 system.

This study was conducted as a retrospective analysis using previously collected clinical data from Suzhou Dushu Lake Hospital. All data were anonymized and contained no personally identifiable information. The research protocol was reviewed and approved by the Medical Ethics Committee of Suzhou Dushu Lake Hospital (Approval No. (2021) Lunyanpi No. 210048), in accordance with the Declaration of Helsinki. Given the retrospective nature of the study and the use of anonymized data, the requirement for individual informed consent was waived by the ethics committee.

### 2.2. Algorithm Principle of VMD

Variational Mode Decomposition (VMD) is an innovative signal processing technique designed to decompose complex signals into sub-signal modes characterized by distinct center frequencies. Introduced by Konstantin Dragomiretskiy and Dominique Zosso in 2014, VMD was developed to overcome the limitations of Empirical Mode Decomposition (EMD) [29]. VMD offers a robust approach to managing nonlinear and non-stationary signals. It operates as an adaptive, non-recursive modal decomposition model, with the core concept being the transformation of the signal decomposition challenge into a variational problem. The intrinsic mode functions (IMFs) are identified by minimizing an energy functional, thereby revealing the signal’s inherent mode functions.

The objective of this VMD is to decompose the original signal into a series of band-limited and compact modal components that are adaptively determined directly from the data. These components possess well-defined center frequencies and minimized bandwidths, which in turn reduce spectral overlap between modes. Additionally, they exhibit favorable local characteristics and reconfigurability.

To circumvent the issue of modal stacking that may occur during signal decomposition, VMD abandons the recursive approach traditionally employed by EMD when calculating IMFs. Instead, VMD employs a fully non-recursive mode decomposition strategy. Compared to conventional signal decomposition algorithms, VMD boasts the benefits of a non-recursive solution and the autonomous selection of the number of modes. In contrast to EMD, VMD delineates each IMF as an amplitude-modulation (AM) frequency modulation function, with a mathematical formulation expressed as:

(1) $$\nu_{k}\left(t\right)=A_{k}\left(t\right)cos\left(\Phi_{k}\left(t\right)\right)$$

where the instantaneous phase $\Phi_{k}(t)$ is a non-monotone decline, that is $\Phi_{k}^{\prime }(t)\geq 0$, the envelope amplitude $A_{k}(t)\geq 0$ of the signal $\nu_{k}\left(t\right)$.

Suppose the original signal s is composed of K components $\nu_{k}\left(t\right)$ each with a limited bandwidth. The central frequency of each IMF is denoted by $\omega_{k}\left(t\right).$ The sum of the bandwidths is minimized, and the aggregate of all the modes equals the original signal. The decomposition process in the VMD algorithm can be interpreted as the process of finding the optimal solution to a variational problem, which can be translated into the formulation and resolution of a variational problem. The detailed steps are as follows:

(1) The solution $\nu_{k}(t)$ is obtained by Hilbert transform [34] and its unilateral spectrum is calculated, and the central band of $e^{-j\omega_{k}t}$ is shifted to the corresponding baseband $\omega_{k}\left(t\right)$ by multiplication with operator $\nu_{k}(t)$:

(2) $${\left[\left(\delta \left(t\right)+\frac{j}{\pi t}\right)*\nu_{k}\left(t\right)\right]e}^{-j\omega_{k}t}$$

(2) Calculate the square norm $L^{2}$ of the demodulating gradient, and estimate the bandwidth of each module component. The constrained variational model is:

(3) $$\left\{\begin{array}{c} \underset{\left\{\nu_{k}\right\},\;\left\{\omega_{k}\right\}}{\min}\left\{{\sum_{k}\left\|{𝜕}_{t}{\left[\left(\delta \left(t\right)+\frac{j}{\pi t}\right)*\nu_{k}\left(t\right)\right]e}^{-j\omega_{k}t}\right\|}^{2}\right\}\\ s.\;t.\;\;\;\;\;\;\;\;\;\;\;\;\;\;\;\;\sum_{k}\nu_{k}=s \end{array}\right.$$

In the above formula, $\left\{\nu_{k}\right\}=\left\{\nu_{1},\nu_{2},\nu_{3},\ldots ,\nu_{k}\right\}$ which represents each IMF component after decomposition, and $\left\{\omega_{k}\right\}=\left\{\omega_{1},\omega_{2},\omega_{3},\ldots ,\omega_{k}\right\}$ is the center frequency of the corresponding component.

To find the optimal solution to the constrained variational problem, we transform the constrained variational problem into an unconstrained variational problem, where the Variational Mode Decomposition introduces Lagrangian multipliers τ(t) and the second order penalty term α. The extended Lagrangian expression is as follows:

(4) $$L\left(\left\{\nu_{k}\right\},\;\left\{\omega_{k}\right\},\;\tau \right)\;=\;\alpha {\sum_{k}\left\|{𝜕}_{t}{\left[\left(\delta \left(t\right)+\frac{j}{\pi t}\right)*\nu_{k}\left(t\right)\right]e}^{-j\omega_{k}t}\right\|}^{2}+{\left\|s\left(t\right)-\sum_{k}\nu_{k}\left(t\right)\right\|}^{2}+\langle \tau \left(t\right),\;s\left(t\right)-\sum_{k}\nu_{k}\left(t\right)\rangle$$

(3) Then use the Alternate Direction Method of Multipliers (ADMM) [26] to alternately update each IMF, and finally obtain the best solution to the original problem. All IMFs can be obtained by the following formula:

(5) $${\hat{\nu }}_{k}^{n+1}\left(\omega \right)=\frac{\hat{s}\left(\omega \right)-\sum_{i\text{≠}k}{\hat{\nu }}_{i}\left(\omega \right)+\frac{\hat{\tau }\left(\omega \right)}{2}}{1+{2\alpha \left(\omega -\omega_{k}\right)}^{2}}$$

where ω represents the frequency, ${\hat{\nu }}_{k}^{n+1}\left(\omega \right)$, $\hat{s}\left(\omega \right)$, $\hat{\tau }(\omega )$ correspond to the Fourier transform of $\nu_{k}^{n+1}\left(t\right)$, $s\left(t\right)$, τ(t), respectively.

(4) ${\hat{\nu }}_{k}^{n+1}\left(\omega \right)$ is the remainder of $\hat{s}\left(\omega \right)-\sum_{i\neq k}{\hat{\nu }}_{i}\left(\omega \right)$ after Wiener filtering. The algorithm re-estimates the center frequency based on the center of gravity of the power spectrum of each IMF. The specific process is:

(1) Initialize $\left\{{\hat{\nu }}_{k}^{1}\right\},\left\{{\hat{\tau }}_{k}^{1}\right\},\left\{{\hat{\omega }}_{k}^{1}\right\}$ and n;

(2) Execution cycle: n = n + 1;

(3) When ω ≥ 0, update ${\hat{\nu }}_{k}$ according to Formula (5);

(4) Update $\omega_{k}$ according to Formula (6);

(6) $$\omega_{k}^{n+1}=\frac{\int_{0}^{\text{∞}}{\omega \left|\nu_{k}^{n+1}\left(\omega \right)\right|}^{2}d\omega }{\int_{0}^{\text{∞}}{\left|\nu_{k}^{n+1}\left(\omega \right)\right|}^{2}d\omega }$$

(5) Update $\hat{\tau }$ according to Equation (7), where β represents noise, when the signal contains strong noise, in order to achieve better denoising effect, β = 0 can be set;

(7) $${\hat{\tau }}^{n+1}\left(\omega \right)={\hat{\tau }}^{n}\left(\omega \right)+\beta \left(\hat{s}\left(\omega \right)-\sum_{k}{\hat{\nu }}_{k}^{n+1}\left(\omega \right)\right)$$

(6) Repeat steps (2) through (5) until iteration stop conditions are met:

(8) $$\sum_{k}{\left\|{\hat{\nu }}_{k}^{n+1}-{\hat{\nu }}_{k}^{n}\right\|}_{2}^{2}/{\left\|{\hat{\nu }}_{k}^{n}\right\|}_{2}^{2}\text{<}\varepsilon$$

where ε is the judgment accuracy, and the K modal components are output after the conditions are satisfied.

### 2.3. Arithmetic Optimization Algorithm (AOA)

The Arithmetic Optimization Algorithm (AOA) is a new optimization method inspired by arithmetic operations, proposed by scholars such as Abualigah in 2021 [35]. This algorithm ingeniously simulates the dynamic interaction process of four basic operations (addition, subtraction, multiplication, and division) as a search mechanism, thereby constructing a solution space exploration framework for optimization problems. Compared with traditional heuristic algorithms, the AOA not only has strong global search capabilities but also effectively balances the weights of the exploration and exploitation stages through adaptive parameter adjustment, thus significantly improving the convergence speed and avoiding the risk of falling into local optima. In addition, AOA has the potential for parallel computing and is suitable for solving various complex optimization problems.

The design concept of the AOA algorithm embodies the intuition and simplicity of mathematical operations, resulting in fewer parameters, easy implementation, and high computational efficiency. Moreover, due to its structure that naturally supports parallel processing, the AOA shows good scalability and flexibility when dealing with large-scale complex optimization problems. This algorithm is applicable to various fields, including but not limited to engineering design optimization, machine learning model tuning, image and signal processing, combinatorial optimization problems, and feature selection. Its efficiency and stability demonstrated in practical applications have promoted the development trend of intelligent optimization algorithms, making it an increasingly favored tool among researchers and engineers.

The AOA first randomly initializes a set of candidate solutions in the solution space. The position of each solution corresponds to a potential solution to the optimization problem, and its quality is evaluated through a fitness function. The level of the fitness value directly reflects the quality of the solution, and the algorithm continuously optimizes the distribution of these solutions through iteration.

In the search process, the AOA adopts a dynamic update mechanism based on arithmetic operations, combining the two stages of exploration and exploitation to balance the search process between global and local. The exploration stage expands the coverage of solutions through random search to avoid falling into local optima, while the exploitation stage finely adjusts the individual positions near the current optimal solution to improve convergence accuracy. The search strategy is dynamically adjusted by control parameters to adapt to the characteristics of different optimization problems.

After each iteration, the population re-evaluates the quality of the solutions according to the fitness function and updates the current optimal solution. Through continuous optimization and adjustment, the AOA can effectively approach the global optimal solution while maintaining population diversity and enhancing search capabilities. This optimization strategy based on arithmetic operations enables the AOA to have high computational efficiency, stability, and robustness when dealing with complex optimization problems.

In the solution space, the AOA initializes a population containing multiple candidate solutions, where each individual is represented by a D-dimensional vector as a potential solution to the optimization problem. To evaluate the quality of these solutions, the AOA uses a predefined fitness function to calculate the fitness value of each individual, which measures how close the solution is to the optimal solution in the search space.

Fitness evaluation provides the optimization direction for the algorithm. By comparing the fitness values between individuals, the AOA dynamically adjusts the search strategy to balance global search and local exploitation. With the iteration of the algorithm, the population is also continuously updated, making individuals gradually converge to the optimal solution while maintaining diversity to avoid falling into local optima, thereby improving search efficiency and optimization accuracy. The AOA controls the search process through arithmetic operations. Compared with Particle Swarm Optimization (PSO) and Genetic Algorithm (GA), it features fewer parameters and more direct calculation, and usually performs better in terms of convergence speed and global search performance [36].

The specific process of the AOA is as follows:

(1) Population parameter initialization

The optimization process first needs to set basic population parameters to formulate a preliminary plan for the entire search process. Parameter initialization defines the range of the search space, the number of candidate solutions, the maximum number of iterations, and the dynamic control factors used to balance global search and local exploitation, providing basic conditions for subsequent searches.

First, the AOA will generate a randomly distributed population with uniform distribution. The initial population can be obtained by the following formula:

(9) $$X\left(i,j\right)=Rand\times \left(Ub-Lb\right)+Lb$$

Among them, Ub is the upper bound, Lb is the lower bound, Rand is a random number between [0, 1], and X(i, j) is the position of the i-th solution in the j-th dimensional space.

(2) Math Optimizer Accelerated function

The Math Optimizer Accelerated (MOA) function in the Arithmetic Optimization Algorithm is a key mechanism for dynamically adjusting the search strategy. This function is designed to balance the two stages of global exploration and local exploitation, thereby maintaining a high degree of search dispersion in the early stage and gradually reducing the search step size in the later stage to achieve fine optimization. The calculation formula of MOA is generally expressed as:

(10) $$MOA(t)=Min+\frac{t\times (Max-Min)}{T}$$

Among them, Min and Max represent the minimum and maximum values of the acceleration function, respectively; T is the maximum number of iterations, and *t* is the current number of iterations. This function shows a linear growth trend during the iteration process. When the value is low in the early stage, it prompts the algorithm to use operators with high dispersion such as multiplication or division to achieve global exploration. As t increases, the value of MOA gradually increases, and the algorithm tends to use operators such as addition or subtraction to reduce the search step size and enhance the local exploitation ability.

A random number R1 between [0, 1] is used to control the calculation stage of the algorithm: when R1 < MOA(t), the function enters the global exploration stage; otherwise, it enters the local exploitation stage.

This strategy based on the mathematical function accelerator enables AOA to adaptively switch from rough search to fine optimization, effectively improving the convergence speed and reducing the risk of falling into local optima. Through the dynamic adjustment of MOA, the algorithm ensures the breadth of global search while being able to conduct detailed exploration of the solution space in the later stage, thereby improving the solution accuracy and overall performance.

(3) Global exploration of the algorithm

In the initial stage, a large-step update strategy is adopted to achieve global exploration, ensuring that the search process can extensively traverse the solution space, thus avoiding falling into local optima early. At this stage, a multiplication or division strategy is chosen based on the random number R2. Both strategies have high dispersion, which is beneficial for solutions to explore in the algorithm space. The calculation formulas are as follows:

(11) $$X(t+1)\;=\left\{\begin{array}{c} X_{b}\;(t)÷(MOP+\varepsilon )\times ((Ub-Lb)\times \mu +Lb)\;\;,R_{2}\;<0.5\\ X_{b}\;(t)\times MOP\times ((Ub-Lb)\times \mu +Lb),otherwise \end{array}\right.\;$$

X(t + 1) is the position of the next-generation particle, X_b_(t) is the position of the particle with the best current fitness, μ is the search process control coefficient (generally set to 0.5), ε is a minimum value, and MOP is the Math Optimizer Probability, whose calculation formula is as follows:

(12) $$MOP(t)=1-{\left(\frac{t}{T}\;\right)}^{\frac{1}{\alpha }}$$

Wherein, MOP(t) is the current Math Optimizer Probability, and α is the iteration sensitivity coefficient. A higher value of α means that the number of iterations has a greater impact on MOP(t). In the early stage of the algorithm (i.e., when t is small), the value of MOP is relatively high, making the update process tend to use multiplication or division operators. This strategy has high dispersion, which is conducive to global search. As t increases, the value of MOP gradually decreases, thereby reducing the search step size and enhancing the local exploitation ability. This mechanism effectively prevents the algorithm from falling into local optima prematurely and improves the overall convergence accuracy and stability.

(4) Local exploitation of the algorithm

In the later stage of the search, to refine the local search, the update step size is gradually reduced to make precise adjustments to the current better solutions. At this stage, local exploitation is mainly carried out through addition and subtraction strategies. Both strategies have significant low dispersion, which makes it easy to approach the target and helps the algorithm find the optimal solution faster. Their calculation formulas are as follows:

(13) $$X(t+1)\;=\left\{\begin{array}{c} X_{b}\;(t)-MOP\times ((Ub-Lb)\times \mu +Lb)\;,R_{3}\;<0.5\\ X_{b}\;(t)+MOP\times ((Ub-Lb)\times \mu +Lb),otherwise \end{array}\right.\;$$

Wherein, R_3_ is a random number within [0, 1].

(5) Main loop of the algorithm (iteration process)

In the main loop, the algorithm continuously iteratively updates the state of candidate solutions and dynamically adjusts the search strategy according to the current number of iterations. First, in each iteration, the fitness values of all candidate solutions in the current population are calculated to determine the global optimal solution. Subsequently, based on the current values of the pre-set Math Optimizer Accelerated (MOA) function and Math Optimizer Probability (MOP), the algorithm switches between global exploration and local exploitation. After calculating the fitness of the candidate solutions, the current optimal solution is found, the global optimal solution is updated, and the boundaries are updated. The main loop terminates when the maximum number of iterations is reached or the convergence criterion is met, ensuring that the algorithm can achieve efficient and stable convergence while fully exploring the solution space.

Nonlinear MOA scheduling: slow in the early stage and steep in the later stage. In the early stage, more multiplication/division operations are used to achieve wide-range exploration; in the later stage, addition/subtraction operations are used to refine neighborhood search.

Fitness function: MEE (Mean Envelope Entropy). For each IMF, the normalized envelope is obtained through Hilbert transform, the entropy is calculated, and the average is taken and minimized. A small MEE indicates that the components are sparser and closer to the dominant cardiac cycle.

### 2.4. Algorithm Improvement and Overall Framework

#### 2.4.1. Improvements

In practical applications, AOA may face problems such as insufficient solution accuracy, unsatisfactory convergence speed, and being easily trapped in local optimal solutions. To address these challenges, our work proposes improved position update rules and a scheme to adjust the optimizer probability in the algorithm, so as to enhance the exploration and exploitation capabilities of the algorithm. In addition, a set of mechanisms is designed to prevent the algorithm from falling into local optima, thereby improving the overall solution effect and the robustness of the algorithm.

(1) Position update strategy

In the Arithmetic Optimization Algorithm, the design of the position update strategy is crucial. Improper position update strategies may cause the algorithm to converge prematurely to local optimal solutions, reduce the global search capability, and affect the optimization effect. In addition, insufficient population diversity may also make it difficult for the algorithm to jump out of local optimal solutions, affecting the global search capability.

To improve this, the Tent chaotic mapping initialization strategy and population mutation strategy are introduced. The Tent Map is a piecewise linear chaotic mapping function, named for its image shape resembling a tent. It is widely used in chaotic encryption systems, such as image encryption, generation of chaotic spread spectrum codes, etc. The definition of the Tent chaotic mapping is as follows:

(14) $$X_{n+1}=\left\{\begin{array}{c} \frac{X_{n}}{\alpha },0\leq X_{n}<\alpha \\ \frac{1-X_{n}}{1-\alpha },\alpha \leq X_{n}\;\leq 1\; \end{array}\right.\;\;\;$$

Wherein, the parameter *α* satisfies 0 < *α* < 1.

**Figure 3:**
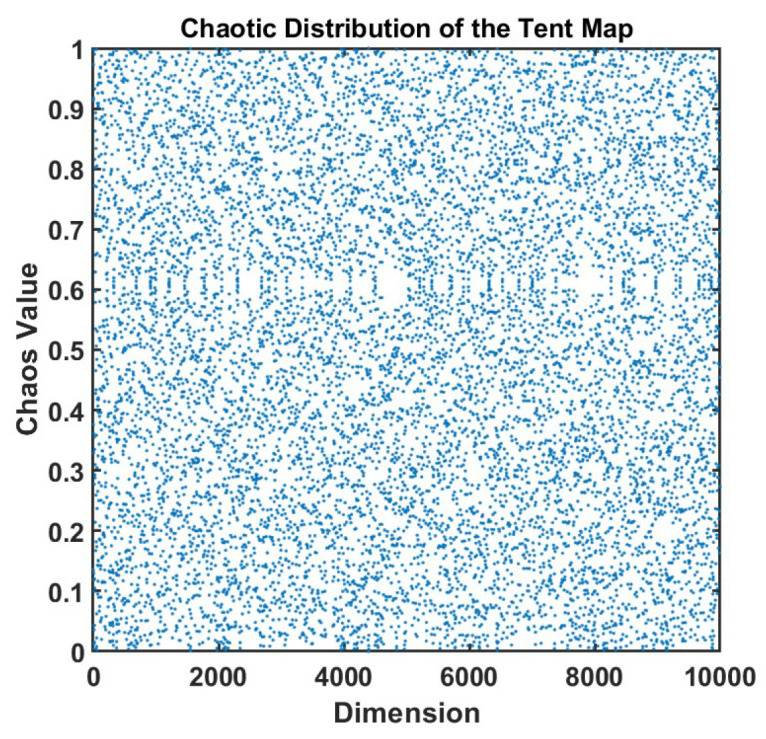
The Tent chaos mapping used in this work produces chaotic values.

As shown in Figure 3, in the scatter plot of the chaotic mapping, the horizontal axis represents the dimension and the vertical axis represents the chaotic value. The data points in the figure are evenly distributed without obvious patterns, showing typical chaotic characteristics, which indicates that the mapping function has strong randomness and ergodicity.

Compared with traditional random initialization, chaotic mapping can significantly enhance the global search capability and convergence accuracy of optimization algorithms. Its ergodicity and pseudo-randomness enable initial individuals to be evenly distributed in the search space, avoiding local concentration and reducing the risk of falling into local optima. Meanwhile, the non-periodic characteristic enhances population diversity, which helps achieve more comprehensive global exploration in the early stage of search and improve the overall optimization efficiency.

In addition, the algorithm adds a population mutation strategy during each population position update to further enhance the search capability and the ability to jump out of local optima. By introducing chaotic disturbance or adaptive mutation mechanisms in the iteration process, individuals make fine adjustments on the basis of their current positions, enabling them to maintain high exploration capability in the search space and avoid premature convergence. Different from the traditional fixed mutation rate, this dynamic mutation method can appropriately adjust the mutation amplitude according to the search progress, further improving the algorithm’s ability to escape local optima. The formula is as follows:

(15) $${X(t+1)=r}_{1}{*(X}_{b}{(t)-X(t))+(1-r}_{1}{)*(X}_{rand}-X(t))$$

Wherein, r_1_ is a random number within [0, 1], and X_rand_ is a random individual within the range of the current population.

(2) Mathematical Optimizer Accelerated Function (MOA)

The MOA function is the core control mechanism of the AOA. It drives the algorithm performance by dynamically adjusting the balance between exploration and exploitation. Its core functions are reflected in: dynamically coordinating the switch between global search and local optimization, using a linearly increasing threshold (MOA value) to favor global exploration (multiplication and division operations) in the early stage, and gradually shifting to local exploitation (addition and subtraction operations) in the later stage; optimizing resource allocation, and adjusting parameter weights in coordination with the Math Optimizer Probability (MOP) to ensure that the algorithm takes both diversity (avoiding prematurity) and convergence (approaching the optimal solution) into account during iteration. However, the linear change strategy of MOA lacks adaptability to complex problems, which may lead to insufficient early exploration or delayed later exploitation; at the same time, its strong dependence on randomness and parameter sensitivity are prone to cause local optimal traps, which need to be improved through nonlinear reconstruction, adaptive mechanisms, etc., to enhance robustness. For this reason, this study proposes a nonlinear MOA acceleration function, and its formula is as follows:

(16) $$\mathrm{OMOA}(t)=(\frac{t}{T}{)}^{2e^{\left(1-\frac{t}{T}\right)}}$$

**Figure 4:**
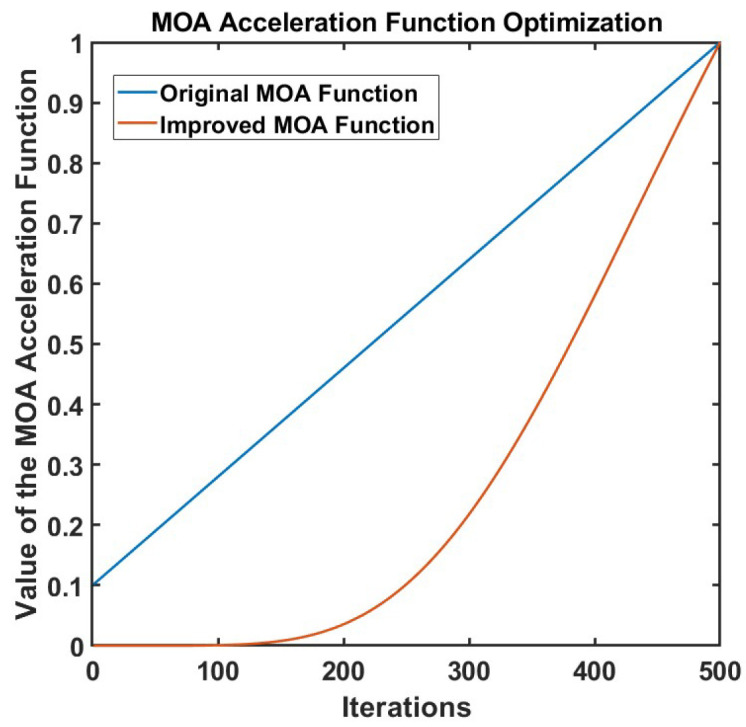
Comparison of optimization effects of MOA mathematical optimizer accelerated functions.

The comparison effect between the improved MOA function and the original MOA function is shown in Figure 4. Compared with the linear MOA function, the nonlinear MOA function proposed in this work shows better performance in the algorithm. It can be seen from the figure that as the number of evolutionary iterations gradually increases, the linear MOA function grows at a constant rate, while the nonlinear MOA function grows more slowly in the early stage. This effectively avoids the local optimal trap caused by too fast convergence in the early stage of the search, enabling the algorithm to have stronger global exploration ability. With the deepening of iterations, the nonlinear MOA function accelerates its growth and reaches the same final value as the linear MOA in the later stage, thereby enhancing the local search ability of the algorithm and improving the convergence accuracy and optimization efficiency. This improvement allows the algorithm to dynamically adjust the search step size at different stages, improving the local convergence accuracy while maintaining the global search ability, making the optimization effect more stable and reliable.

#### 2.4.2. Algorithm Framework

When applying VMD to process signals, the decomposition level K and the penalty factor α are key parameters that affect the decomposition effect. If K is set too small, it may lead to insufficient signal decomposition and incomplete information extraction; while an excessively large K tends to cause over-decomposition, increasing redundancy and computational complexity. Meanwhile, the selection of the penalty factor directly affects the bandwidth of Intrinsic Mode Function (IMF) components, thereby influencing the accuracy of signal reconstruction.

The traditional method of manually setting parameters is inefficient and difficult to ensure the optimal combination. Although existing studies have attempted to optimize K through the center frequency observation method, this method is cumbersome to operate and cannot optimize α simultaneously. For this reason, this work introduces the improved AOA algorithm to realize the joint adaptive optimization of K and α. AOA can efficiently search for the optimal solution in the continuous parameter space, significantly improving the accuracy and stability of VMD decomposition, while reducing manual intervention and enhancing the overall processing efficiency and the quality of signal feature extraction.

Mean envelope entropy is an indicator used to measure signal sparsity, reflecting the uniformity of the distribution of signal energy or information on the time axis. In this study, the VMD algorithm is used to decompose the magnetocardiographic signal into K intrinsic mode functions (IMFs). If some IMF components contain more noise, their pulse characteristics are not obvious and their periodicity is weak, which will be manifested as reduced sparsity and increased envelope entropy; conversely, if the IMF clearly retains the periodic characteristics of the original signal, its sparsity is stronger and the corresponding envelope entropy is smaller. In the process of the algorithm optimizing VMD parameters [K, α], this work selects Mean Envelope Entropy (MEE) as the fitness function [37]. By minimizing MEE, the parameter optimization problem is transformed into an optimization process where AOA searches for the minimum envelope entropy value in the solution space. Assuming that during the decomposition process, the input signal s(t) is decomposed into K IMFs, the calculation formula of the mean envelope entropy is as follows:

(17) $$\left\{\begin{array}{c} E_{pj}=-\sum_{j=1}^{N}p_{j}lgp_{j}\\ \begin{array}{c} p_{j}=\frac{a\left(j\right)}{\sum_{j=1}^{N}a\left(j\right)}\\ E_{MEE}=\frac{1}{K}\sum_{j=1}^{N}E_{pj} \end{array} \end{array}\right.$$

N is the length of the signal; K Intrinsic mode functions (IMFs) can be obtained through VMD decomposition, and then the normalized envelope signals are acquired after performing Hilbert transform on them; subsequently, the envelope entropy of each IMF is calculated.

The Arithmetic Optimization Algorithm is used to optimize the penalty factor and decomposition level of VMD. The optimization steps are as follows:

(1) Set the value range of VMD parameters and randomly generate a set of initial parameters [K, α];

(2) Perform VMD decomposition according to the generated parameter set to obtain the corresponding IMF components;

(3) After calculating the mean envelope entropy, select the set of parameters with the smallest envelope entropy as the current optimal solution, and calculate the corresponding MOA function at the same time;

(4) Determine whether R1 is less than the MOA function. If yes, perform global search (step 5); otherwise, perform local exploitation (step 6);

(5) If R_2_ < 0.5, perform division search operation; otherwise, perform multiplication search operation;

(6) If R_3_ < 0.5, perform subtraction exploitation operation; otherwise, perform addition exploitation operation;

(7) If the maximum number of iterations is reached, output the current optimal parameters; otherwise, return to step (2) to continue the iteration.

To sum up, the mathematical model of the parameters can be summarized as follows:

(18) $$\left\{\begin{array}{c} minE_{MEE}=f\;\left(K,\alpha \right)\\ s.t.\;\;K_{min}\text{<}\;K\;\text{<}\;K_{max},\alpha_{min}\text{<}\;\alpha \;\text{<}\;\alpha_{max} \end{array}\right.$$

Wherein, f (K, α) is the average envelope entropy of each IMF calculated by VMD based on the current parameters K and α.

To sum up, the steps of the improved denoising algorithm are as follows:

(1) First, use AOA to adaptively optimize the key parameters of VMD. Specifically, the algorithm calculates the average envelope entropy of the IMFs decomposed under different parameter combinations through iteration, and takes the parameter combination [K, α] with the minimum entropy value as the optimal solution;

(2) Based on the optimized parameters K and α, perform VMD decomposition on the original MCG signal to obtain a series of IMF components;

(3) Conduct spectrum analysis on each decomposed IMF component, and implement differentiated processing according to its central frequency characteristics: IMF components with a central frequency higher than 50 Hz are directly eliminated; IMF components with a central frequency lower than 50 Hz undergo wavelet threshold denoising; the lowest frequency IMF component is subjected to baseline drift correction;

(4) Reconstruct the IMF components processed as above to obtain the final denoised signal.

**Figure 5:**
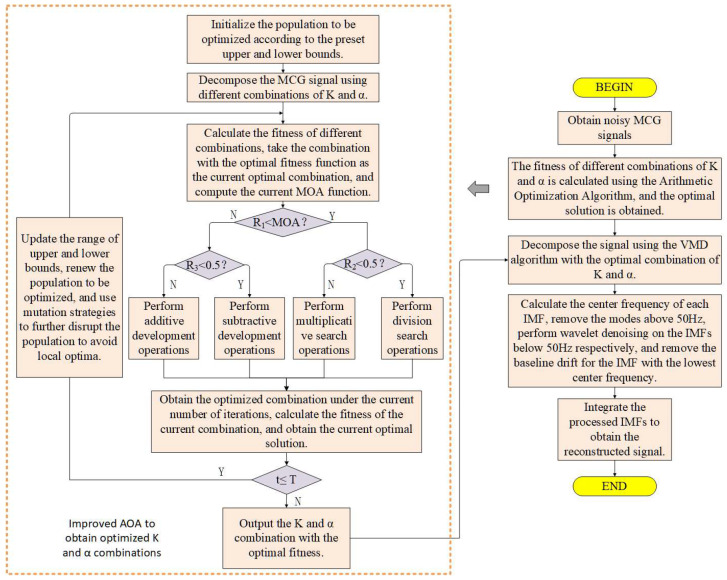
Flowchart of the improved denoising algorithm.

The overall flow chart of the algorithm is shown in Figure 5.

### 2.5. Evaluation Indicators and Statistics

To comprehensively evaluate the denoising performance of different algorithms, four metrics were selected: signal residual analysis, QRS wave amplitude change, spectral entropy, and suppression amount of high and low frequency noise frequencies of the denoised signal to describe the denoising effect of each algorithm. Smaller QRS-wave amplitude changes (e.g., closer to zero), lower spectral entropy, and a greater suppression of high and low frequency noise indicate superior denoising performance. Signal residual analysis evaluates the denoising effect visually: if the signal features contain fewer recognizable signal features, it implies better signal retention and consequently, a stronger denoising effect. The calculation formulas of the three are as follows:

High-frequency noise frequency suppression amount (HFNSA):

(19) $$\Delta P_{high\;}=10log_{10}\;(P_{high\_raw}\;/P_{high\;})$$

Low-frequency noise frequency suppression amount (LFNSA):

(20) $$\Delta P_{low}=10log_{10}\;(P_{low\_raw}\;/P_{low\;})$$

Among them, P_high_ and P_low_ represent the power of high-frequency and low-frequency noise in the denoised signal, respectively, while P_high_raw_ and P_low_raw_ represent the power of high-frequency noise (greater than 40 Hz) and low-frequency noise (less than 0.5 Hz) in the original signal.

QRS wave amplitude change (QRSA):

(21) $$\Delta A_{QRS}\;=\frac{A_{processed}\;-A_{raw}\;\;}{A_{raw}\;}\times 100\%$$

Wherein, A_raw_ is the average amplitude of the QRS complex in the original signal. A_processed_ is the average amplitude of the QRS complex in the denoised signal.

Spectral entropy:

(22) $$H=-\sum_{i=1}^{N}\frac{PSD_{i}}{\sum_{j=1}^{N}PSD_{j}\;}*\;\;\;log_{10}\;(\frac{PSD_{i}\;}{\sum_{j=1}^{N}\;PSD_{j}}\;+\varepsilon )$$

Wherein, PSD_i_ is the power spectral density of the i-th frequency component, $\frac{PSD_{i}}{\sum_{j=1}^{N}PSD_{j}\;}$ is the normalized energy distribution (probability), and ε is a very small positive number used to avoid taking the logarithm of zero.

## 3. Results

### Experimental Results

**Figure 6:**
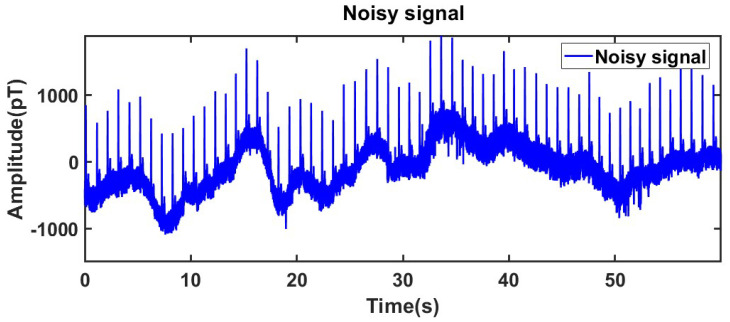
Collected noisy signals.

To evaluate the practical performance of the proposed method, continuous MCG detection of the human heart was performed for 60 s under open and low-noise conditions, with a sampling frequency of 1 kHz. The measured signal is shown in Figure 6. In the process of population optimization, the populations size per iteration is set to 60, and the maximum number of iterations is set to 50. The ranges of parameters K and α are set to K ∈ [4, 10] and α ∈ [500, 3000].

**Figure 7:**
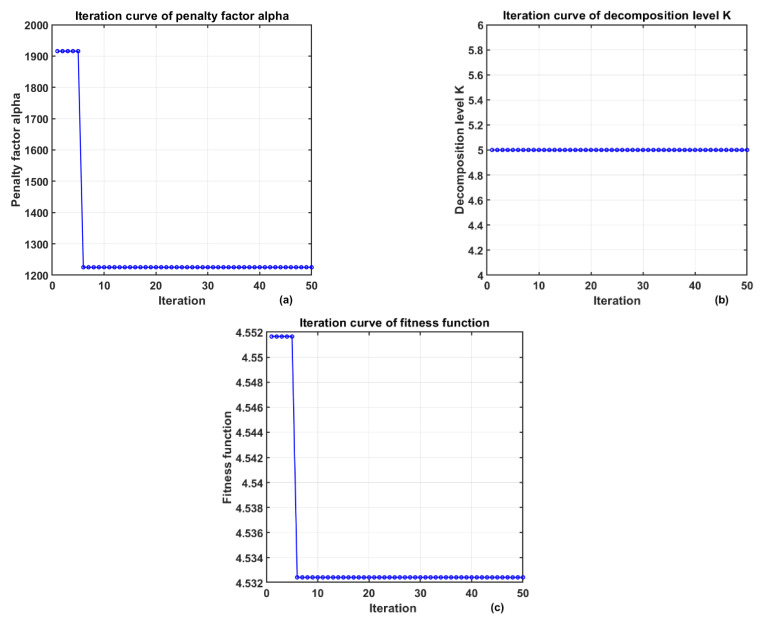
The change in VMD parameters optimized by AOA. (**a**) Iteration Curve of the Penalty Factor (**b**) Iteration Curve of the Decomposition Level (**c**) Iteration Curve of the Fitness Function.

In the signal denoising experiment, the optimization curve of the algorithm is shown in Figure 7. At this time, K = 5, α = 1335.6098, and the obtained envelope entropy EMEE = 4.5563. The signal was decomposed by VMD based on the above parameter combination.

**Figure 8:**
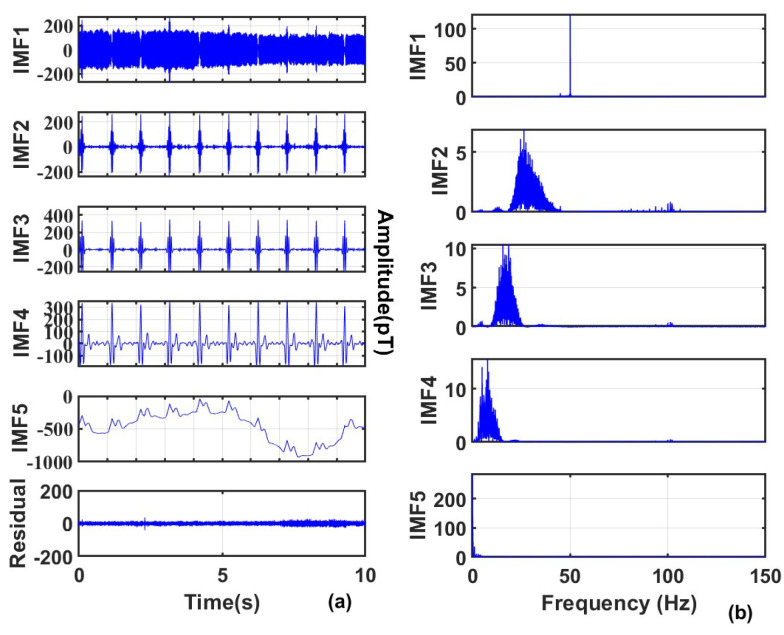
(**a**) Results of signal decomposition; (**b**) Frequency domains of each component.

To more intuitively show the denoising effect, this work selected the real-time MCG data of the human body from 0 to 10 s for analysis, and the decomposition results are shown in Figure 8.

**Figure 9:**
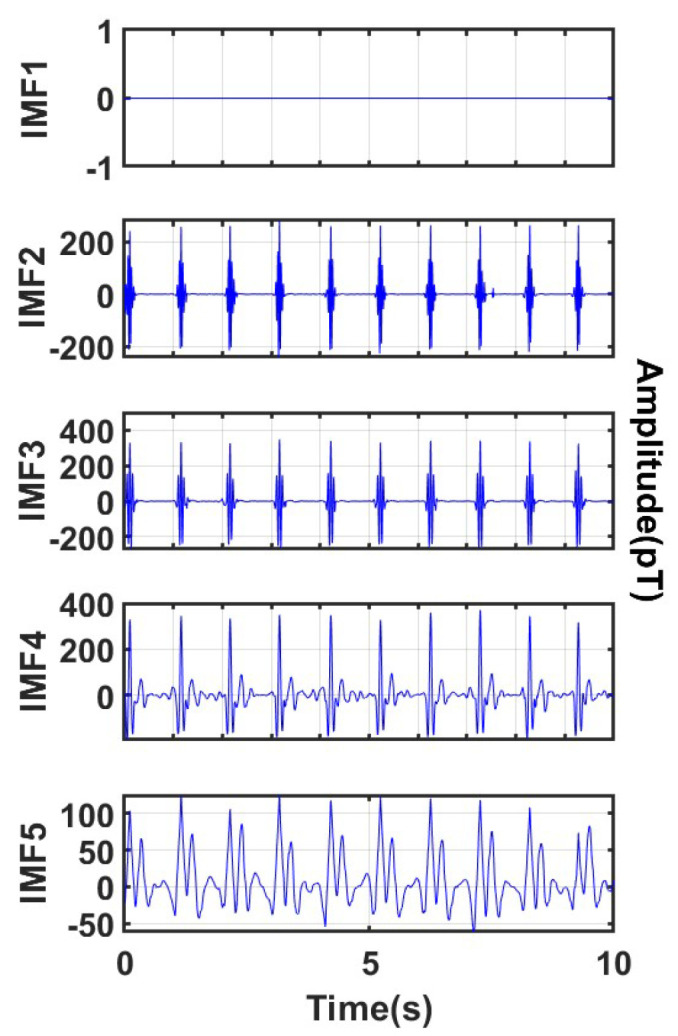
Processing of IMFs obtained by disaggregation.

**Figure 10:**
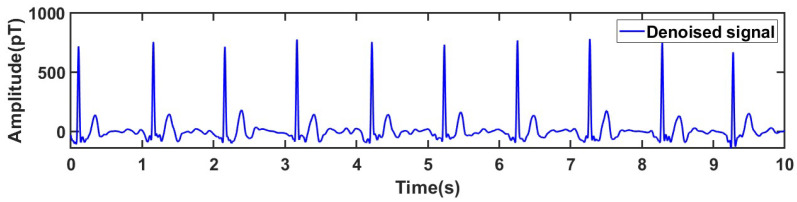
Reconstructed real MCG signal.

It can be clearly seen that through the optimal parameter combination, the signal can be well decomposed into the same mode with different frequencies, which is convenient for subsequent threshold processing. Among them, IMF1 has a central frequency higher than 60 Hz, so it is completely removed; IMF 2 to IMF 5 have central frequencies lower than 60 Hz, and wavelet denoising is performed on each IMF; IMF 5 has a central frequency lower than 0.5 Hz, so additional baseline drift noise removal is performed on IMF 5, and the removal result is shown in Figure 9. Finally, the denoised signal is obtained by reconstruction, as shown in Figure 10.

The results demonstrate that the proposed algorithm effectively removes baseline drift while preserving the key waveform characteristics of the MCG signal. The processed signal shows high smoothness and minimal fluctuation deviation, further confirming the reliability and practicability of the algorithm.

**Figure 11:**
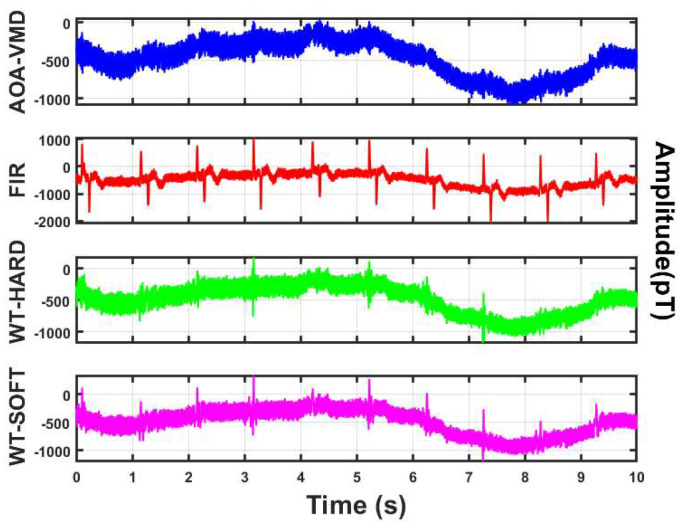
Comparison of signal residuals obtained by different algorithms.

Through signal residual analysis, as shown in Figure 11, it can be seen that the signal residual waveform of the algorithm in this work is relatively smooth with small amplitude, and there are no obvious periodic features or sharp QRS wave characteristics (such as heartbeat peaks). This indicates that the algorithm has removed most of the noise, and the residual is close to random noise. However, the signal residuals obtained by other algorithms show more or less periodic sharp waves, i.e., the R-wave characteristics of the signal. This further proves the reliability of the proposed algorithm.

**Figure 12:**
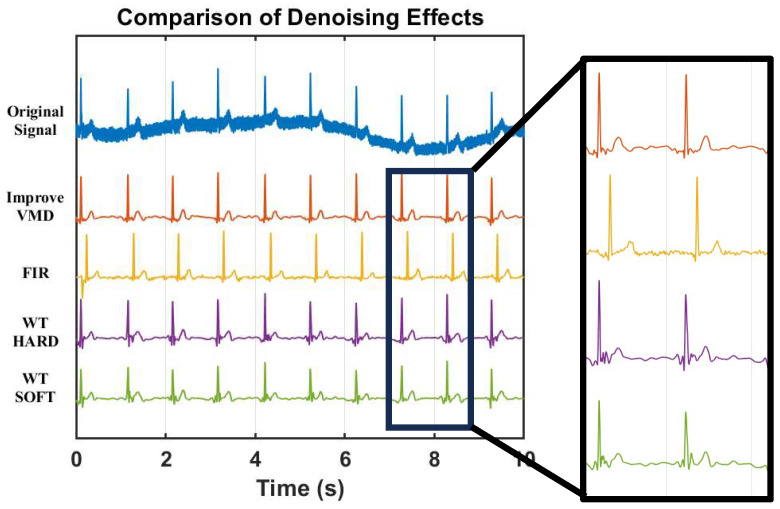
Comparison of the effect of different algorithms on real MCG signal after processing.

The method adopted in this work is compared with other algorithms, and a denoising experiment is conducted on the measured MCG data. The denoising effect is shown in Figure 12.

It is found through comparative experiments that the signals processed by other algorithms still have a lot of residual noise, and the amplitude of the R peak is reduced after denoising. The reduction degree of the soft threshold processing is more obvious than that of the hard threshold.

**Figure 13:**
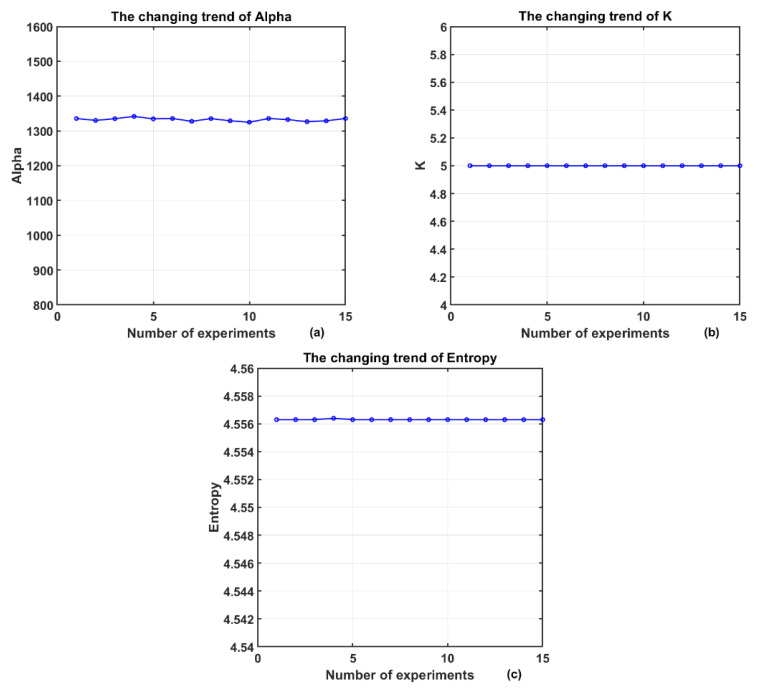
Results of multiple experiments on the same signal: (**a**) The changing trend of Alpha; (**b**) The changing trend of K; (**c**) The changing trend of Entropy.

To verify the stability of the algorithm and avoid falling into local optimal solutions, this work conducts multiple independent tests on the same segment of MCG signals. The test results are shown in Figure 13.

The decomposition level K is stably maintained at 5, while the penalty factor α fluctuates within the range of 1320 to 1340. The mean envelope entropy remains between 4.5563 and 4.5564 with extremely small fluctuations, indicating that the algorithm exhibits good stability across multiple runs.

The comprehensive performance of different algorithms on multiple sets of signals is summarized in Table 1. The proposed method outperforms FIR and wavelet method in both high-frequency and low-frequency suppression. It produces smaller QRS amplitude changes, achieves higher fidelity; the spectral entropy is lower, making the signal purer and more stable.

It is also pointed out in the work that other algorithms still have more residual noise after processing, and the amplitude of the R peak is generally reduced, with the soft threshold showing a more obvious weakening effect, which is consistent with the improvement direction of the above three types of indicators.

## 4. Discussion

The proposed denoising method outperforms traditional techniques in QRS amplitude preservation, spectral entropy reduction, and noise suppression. It improves the signal-to-noise ratio (SNR) of MCG while ensuring signal integrity, thus providing reliable data support for accurate cardiac diagnosis. These results highlight both the novelty and application potential of the algorithm.

In clinical workflows, denoised MCG can directly support (1) R-wave detection and cycle averaging, (2) spatial interpolation and isomagnetic map plotting, and (3) magneto-electric inversion. For R-wave detection, a 5–25 Hz light bandpass filter and Pan–Tompkins’ adaptive threshold yields a robust R sequence. Subsequent R-R alignment and weighted averaging (weights ∝ segment SNR) enhance cross-beat alignment and improve the clarity of butterfly diagrams [38]. For spatial analysis, bicubic spline interpolation on a 5–10 mm grid is validated with leave-one-channel cross-validation to ensure interpretability. For inversion, the Biot–Savart forward model with Tikhonov or TV regularization is solved via conjugate gradient with Laplacian prior, producing stable time-consistent pseudo-current densities.

The algorithmic complexity of AOA–VMD–WT is about O (P·I·K·N log (N)), which is suitable for offline processing on standard workstations. Near-real-time performance can be achieved through multi-channel parallelism, FFT batch processing, and warm starts by caching previously optimized (K, α). Although optimization overhead remains the main bottleneck, lightweight proxy predictors combined with incremental VMD and parallelism can reduce delay for quasi-real-time use.

Regarding generalization, sensor sensitivity, Dewar geometry, and array configuration affect the spectral profile and optimal thresholds. The empirical cutoffs of 0.5 Hz and 60 Hz are reasonable, but tuning within 0.3–0.7 Hz and 40–60 Hz is recommended. Variations in heart rate and chest wall anatomy also require robust thresholds and hierarchical statistics. Looking forward, emerging OPM sensors—characterized by room-temperature operation, flexible layouts, and enhanced low-frequency sensitivity [39,40]—are expected to align well with the proposed method. Subsequent studies may explore sensor–algorithm co-optimization, such as adaptive frequency division tuned to OPM response characteristics, or hybrid denoising frameworks leveraging OPM’s high sensitivity. This integration will pave the way for portable, high-resolution MCG systems suitable for both clinical and point-of-care settings.

## 5. Conclusions

In summary, focusing on the usability of magnetocardiography measurements in a non-magnetic shielding environment, this work adaptively optimizes the key parameters of VMD through AOA, effectively reducing the sensitivity of decomposition to (K, α). Under the multi-stage strategy of frequency-aware modal disposal, wavelet thresholding, and baseline correction, a favorable balance between high- and low-frequency suppression and waveform fidelity is achieved. The high-frequency suppression is improved by approximately +8–+15 dB, the low-frequency suppression by about +2–+8 dB, and the spectral entropy is reduced by about 0.1–0.6, with better preservation of QRS amplitude. Thus, it can more smoothly connect key downstream modules such as R-wave detection and cycle averaging, spatial isomagnetic maps, and magneto-electric inversion, showing consistent advantages in visual inspection of the time domain, frequency domain, and residual spectrum. For future applications, on the one hand, the process can be advanced to real time or near real time through mechanisms such as GPU parallelism, incremental VMD, and lightweight proxy regressors; on the other hand, remote reference channels and body motion sensors should be introduced to achieve stronger adaptive suppression of multi-source low-frequency artifacts such as those from elevators, gait, and bed movement. At the same time, learning-based enhancement schemes constrained by physical priors (such as regressing (K, α) with a small network or performing end-to-end residual noise suppression, combined with uncertainty estimation and interpretability analysis) should be explored to further improve stability and extrapolation ability while maintaining engineering interpretability.

## Figures and Tables

**Table 1:** Comparison of evaluation parameters of different denoising methods.

Number	Evaluation Metrics	Algorithm in This Work	FIR	WT (Soft)	WT (Hard)
1	HFNSA	35.4713	23.6793	24.1046	28.0061
	LFNSA	32.4057	30.1879	32.3746	32.3482
	QRSA	−67.0738	−69.5050	−80.8123	−72.5422
	Spectral entropy	2.8685	2.9714	2.9552	3.0115
2	HFNSA	38.5276	26.4182	29.2382	31.7689
	LFNSA	32.6069	26.1851	27.0406	27.0188
	QRSA	−37.8309	−40.8309	−46.7860	−59.7398
	Spectral entropy	2.9151	2.9765	3.0052	3.0193
3	HFNSA	30.3080	18.0546	21.0947	23.3532
	LFNSA	34.6097	18.6195	19.4814	19.4843
	QRSA	−7.8411	−16.7404	−17.0152	−30.5824
	Spectral entropy	2.6245	2.8401	2.7573	2.7550
4	HFNSA	42.4833	28.8521	34.3513	36.4725
	LFNSA	36.4496	34.4625	32.9787	32.9545
	QRSA	−78.2636	−79.1500	−83.3845	−88.2463
	Spectral entropy	2.1942	2.7885	2.5735	2.7566
5	HFNSA	25.1489	19.4292	18.0745	20.8674
	LFNSA	27.3942	19.7790	20.8996	20.8991
	QRSA	−14.7840	−19.3083	−25.9938	−40.4591
	Spectral entropy	2.8356	2.9622	2.9542	2.9984

## Data Availability

The data that has been used is confidential.
